# How electromagnetic fields can influence adult stem cells: positive and negative impacts

**DOI:** 10.1186/s13287-016-0312-5

**Published:** 2016-04-18

**Authors:** Aleksandra Maziarz, Beata Kocan, Mariusz Bester, Sylwia Budzik, Marian Cholewa, Takahiro Ochiya, Agnieszka Banas

**Affiliations:** Laboratory of Stem Cells’ Biology, Department of Immunology, Chair of Molecular Medicine, Faculty of Medicine, University of Rzeszow, ul. Kopisto 2a, 35-310 Rzeszow, Poland; Centre for Innovative Research in Medical and Natural Sciences, Faculty of Medicine, University of Rzeszow, ul. Warzywna 1a, 35-310 Rzeszow, Poland; Department of Biophysics, Faculty of Mathematics and Natural Sciences, University of Rzeszow, ul. Pigonia 1, 35-310 Rzeszow, Poland; Division of Molecular and Cellular Medicine, National Cancer Center Research Institute, 5-1-1 Tsukiji, Chuo-ku, 104-0045 Tokyo Japan

## Abstract

The electromagnetic field (EMF) has a great impact on our body. It has been successfully used in physiotherapy for the treatment of bone disorders and osteoarthritis, as well as for cartilage regeneration or pain reduction. Recently, EMFs have also been applied in in vitro experiments on cell/stem cell cultures. Stem cells reside in almost all tissues within the human body, where they exhibit various potential. These cells are of great importance because they control homeostasis, regeneration, and healing. Nevertheless, stem cells when become cancer stem cells, may influence the pathological condition. In this article we review the current knowledge on the effects of EMFs on human adult stem cell biology, such as proliferation, the cell cycle, or differentiation. We present the characteristics of the EMFs used in miscellaneous assays. Most research has so far been performed during osteogenic and chondrogenic differentiation of mesenchymal stem cells. It has been demonstrated that the effects of EMF stimulation depend on the intensity and frequency of the EMF and the time of exposure to it. However, other factors may affect these processes, such as growth factors, reactive oxygen species, and so forth. Exploration of this research area may enhance the development of EMF-based technologies used in medical applications and thereby improve stem cell-based therapy and tissue engineering.

## Background

Many, if not all, tissues of the human body are thought to contain stem cells (called adult stem cells/adult tissue stem cells/progenitor cells) that are responsible for tissue regeneration and repair after injury. Adult stem cells are influenced by many biochemical and biophysical stimuli in their in vivo microenvironment, including fluid shear stress, hydrostatic pressure, substrate strains, trophic factors, the electromagnetic field (EMF), and so forth. Depending on the niche in which they reside, as well as the biochemical and biophysical stimuli, stem cells may differentiate or not into desired tissues [1–3]. These factors are of great importance because dysregulation of tissue regeneration and homeostasis may result in various pathological conditions, cancer being the most extensively described. Several studies have focused on the circumstances that result in adult stem cells becoming cancer stem cells (tumor-initiating cells) that participate in carcinogenesis and metastasis. However, the nature of the interaction between adult and cancer stem cells and the mechanisms underlying the putative transition remain elusive. It is believed that during the initial stage of the pathological process, adult stem cells may be both “heroes” and “villains”.

External environmental factors are commonly known to be simultaneously involved in pathological processes, making the maintenance of homeostasis a difficult challenge. Biophysical stimuli may cause downstream signaling towards pleiotropic processes in adult stem cells.

The EMF is pervasive throughout the environment and, owing to technological developments, seems to have great potential as a therapeutic tool. It has significant effects on cells, tissues, and many processes within organisms and plays an important role in biological processes involving adult stem cells, such as embryogenesis, regeneration, and wound healing [4], as well as in cell migration, DNA synthesis, and gene expression [5–7]. However, the data regarding the influence of the EMF on adult stem cell biology are inconsistent.

Here, we review the current knowledge on the effects of EMFs on adult stem cells. Our goal is to present all available evidence for both the positive (stimulative and prodifferentiative) and negative (carcinogenic) impact of EMFs on stem cell biology.

## Adult stem cells

Adult stem cells compose “a reservoir” of cells at various stages of development and possess the unique ability to self-renew and to differentiate into many types of specialized cells [8]. They play an important role in tissue regeneration and maintenance of homeostasis [1, 2, 9, 10]. Adult stem cells isolated and cultured ex vivo may differentiate under proper conditions and may give rise to multiple lineages in a controlled manner in vitro [9]. The cells can thus be used as an autologous source of cells for treatment of multiple modern-age diseases such as cardiovascular diseases [11], liver disease [12–16], and neurogenerative diseases [17]. What is more, the extracellular vesicles derived from adipose-derived mesenchymal stem cells (ASCs) [18–20] have been of particular interest due to their therapeutic activity.

**Fig. 1:**
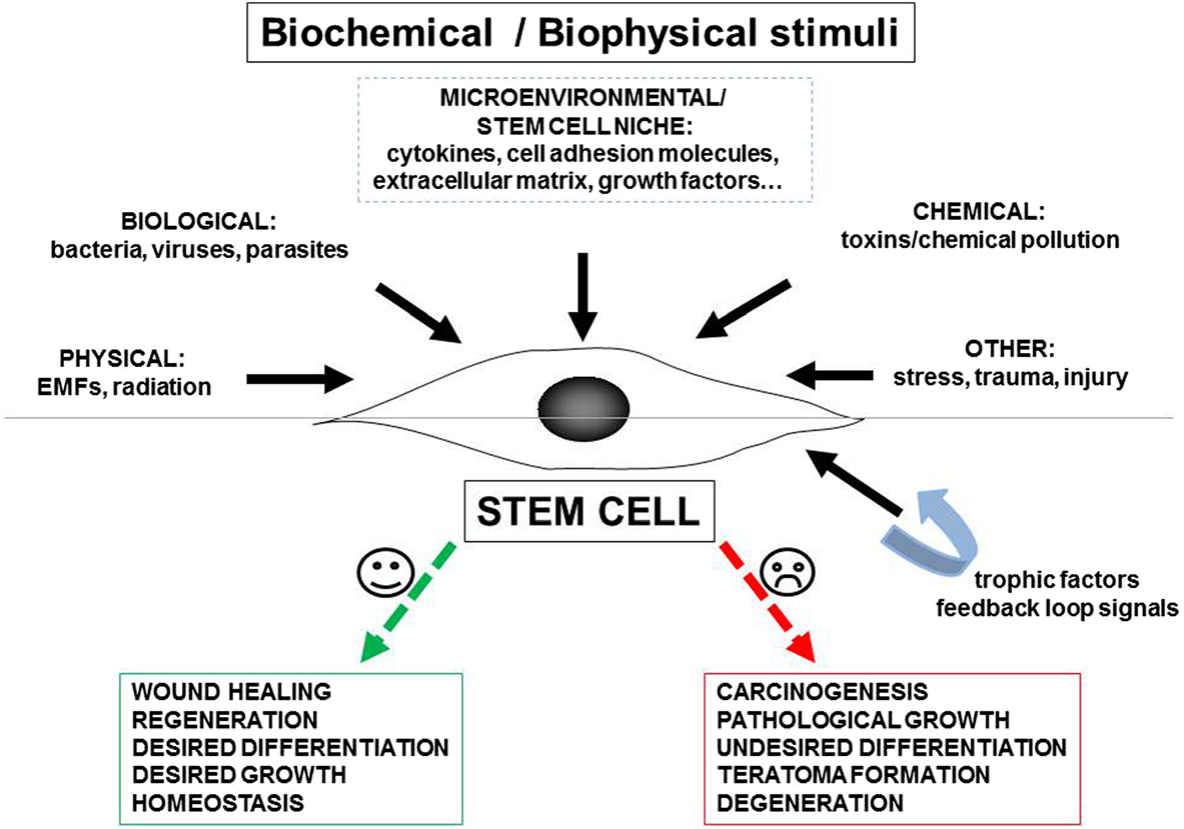
Possible biochemical/biophysical stimuli affecting adult stem cells within the body that lead to physiological or pathological processes. The stimuli may lead towards positive, life-supporting processes (wound healing, regeneration, homeostasis) or negative, life-suppressing processes (carcinogenesis, degeneration). *EMF* electromagnetic field

On the other hand, adult stem cells under the influence of “improper stimuli” may contribute to carcinogenesis and pathological alterations, resulting in many chronic disorders. These stimuli may consist of biochemical and biophysical environmental factors which lead to imbalance in tissues and the stem cell niche. This initiates a cascade of degeneration, destruction, and anti-homeostatic processes, followed by diseases and finally death (Fig. 1).

## The EMF as a therapeutic tool

EMF stimulation has been used successfully for the treatment of bone disorders for many years [5, 21–23]. It is clinically beneficial for bone fracture healing, treatment of osteoarthritis, and pain reduction [23]. The EMF stimulates osteogenesis, increases bone mineral density, decreases osteoporosis, and acts chondroprotectively [6, 23] (Table 1).

**Table 1:** Effects of EMFs with different parameters on stem cell biology

Stem cell type	EMF characteristics	Exposure duration	Differentiation type	Stimulation effects	Reference
Sinusoidal EMF					
BM-MSCs	ELF-EMF Magnetic flux density: 1 mT Frequency: 50 or 100 Hz	Continuous for up to 8 days	Neurogenic	No effects on cell viability Increase in the expression of neuronal markers (NeuroD1, MAP2, NF-L) Stimulation of neural differentiation	Park et al. 2013 [17]
BM-MSCs	ELF-EMF Magnetic flux density: 1 mT Frequency: 50 Hz	Continuous for 12 days	Neurogenic	Inhibition of MSC growth Decrease of the neural stem cell marker expression (nestin) Increase of the neural cell marker expression (MAP2, NeuroD1, NF-L, and Tau)	Cho et al. 2012 [39]
BM-MSCs	ELF-EMF Magnetic flux density: 5 mT Frequency: 15 Hz	Three times a day (45 min every 8 h) for 21 days	Chondrogenic	More compact structure Varied effects on cartilage-specific marker expression (increase in COL II, decrease in COL X, or no impact on aggrecan, SOX9) Higher glycosaminoglycan/DNA content Improvement of chondrogenic differentiation in combination with growth factor treatment	Mayer-Wagner et al. 2011 [23]
BM-MSCs (derived from fetus)	ELF-EMF Magnetic flux density: 20 mT Frequency: 50 Hz	12 h/day for up to 23 days	Osteogenic	Decrease of MSC growth and metabolism No significant effect on MSC differentiation	Yan et al. 2010 [38]
ASCs	EMF Magnetic flux density: 1 mT Frequency: 30/45 Hz (positive differentiation conditions); 7.5 Hz (negative differentiation conditions)	8 h/day	Osteogenic	Alterations in ALP expression level Alterations in osteogenic differentiation level Alterations in the expression of osteogenic markers Enhancement of matrix mineralization	Kang et al. 2013 [6]
ESCs	Low-frequency EMF Magnetic flux density: 5 mT Frequency: 1, 10, and 50 Hz	30 min/day for 3, 5, or 7 days	–	Increase in cell proliferation rate, in a frequency-dependent manner (the highest rate in the 50 Hz group) Alterations in the cell cycle No effect on cell morphology and cell phenotype	Zhang et al. 2013 [35]
Combination of static and sinusoidal EMF					
CSCs	Static MF Magnetic flux density: 10 μT Sinusoidal ELF-EMF Magnetic flux density: 2.5 μT Frequency: 7 Hz (Ca^2+^ ICR)	Up to 5 days	Cardiogenic	Increase in metabolic activity Increase in proliferation rate Increase in the expression of cardiac markers (TnI, MHC, Nkx2.5) Decrease (SMA) or no change (VEGF, KDR) in the expression of vascular markers Alterations in the intracellular calcium distribution	Gaetani et al. 2009 [11]
CSCs/BM-MSCs	Static MF Magnetic flux density: 10 μT Sinusoidal ELF-EMF Frequency: 7 Hz (Ca^2+^ ICR)	For 5 days	Cardiogenic/osteogenic	Upregulation of cardiac markers (TnI, MHC) Downregulation of angiogenic markers (VEGF, KDR) Increase in the expression of osteogenic markers (ALP, OC, OPN) Alterations in plasma membrane morphology accompanied by a rearrangement in actin filaments	Lisi et al. 2008 [43]
Pulsed EMF					
BM-MSCs	Magnetic flux density: 1.1 mT Frequency: 5, 25, 50, 75, 100, and 150 Hz	30 min/day for 21 days	Osteogenic	Alterations in cell morphology Increase in ALP expression and activity Increase in the expression of osteogenic markers (COL I, OC) Stimulation of osteogenic differentiation Enhancement of matrix mineralization	Luo et al. 2012 [7]
BM-MSCs	Magnetic flux density: 1.8–3 mT Frequency: 75 Hz	8 h/day for 14 days	Osteogenic	Acceleration of cell proliferation Alterations in cell cycle Increase in ALP expression level Enhancement of the osteogenic differentiation	Esposito et al. 2012 [45]
BM-MSCs	Time of pulses: 300 μs (repetitive single quasi-rectangular pulses) Magnetic flux density: 0.13 mT Frequency: 7.5 Hz	2 h/day for 14 days	Osteogenic	Time-dependent alterations in cell proliferation rate Stimulation of ALP activity at day 7 Enhancement of early osteogenic genes expression (Runx2/Cbfa1 and ALP) during the mid-stage of osteogenic differentiation	Tsai et al. 2009 [5]
BM-MSCs	Time of bursts: 5 ms Time of pulses: 5 μs Magnetic flux density: 0.1 mT Frequency:15 Hz	Continuous exposure	Osteogenic	Increase of matrix mineralization No effect on ALP activity Upregulation of several osteogenic marker genes (BMP-2, OC, OPG, IBSP, MMP-1, MMP-3) Stimulation of osteogenic differentiation	Jansen et al. 2010 [41]
BM-MSCs/osteoblast-like cells	Time of bursts: 5 ms Time of pulses: 1 μs Magnetic flux density: 0.1 mT Frequency:15 Hz	Continuous exposure	Osteogenic	Increase of cell viability rate No effect on osteo-induction	Kaivosoja et al. 2015 [47]
BM-MSCs	Time of bursts: 4.5 ms Number of pulses: 20 Magnetic flux density: 1.8 mT (increase from 0 to 1.8 mT in 200 μs steps and then decrease to 0 mT in 25 μs steps during each pulse) Frequency: 15 Hz	8 h/day during culture period	Osteogenic, adipogenic, neurogenic	Enhancement of cell proliferation rate Increase of cell densities Alterations of cell cycle progression No effect on the surface phenotype or multilineage differentiation potential	Sun et al. 2009 [21]
BM-MSCs	Time of bursts: 4.5 ms Number of pulses: 20 Magnetic flux density: 1.8 mT (increase from 0 to 1.8 mT in 200 μs steps and then decrease to 0 mT in 25 μs steps during each pulse) Frequency: 15 Hz	8 h/day during the culture period	Osteogenic	Increase in cell proliferation Increase in ALP expression and activity Time-dependent alterations of osteogenic marker expression (BMP-2, Cbfa1, COL I, OC) Enhancement of matrix mineralization	Sun et al. 2010 [33]
BM-MSCs/osteoblast-like cells	Time of bursts: 4.5 ms Number of pulses: 20 Magnetic flux density: 1.6 mT (increase from 0 to 1.6 mT in 200 μs steps and then decrease to 0 mT in 25 μs steps during each pulse) Frequency: 15 Hz	8 h/day	Osteogenic	Surface-dependent decrease in cell number Increase in OPG expression level	Schwartz et al. 2009 [37]
BM-MSCs/ASCs	Number of pulses: 10 Time of pulses: 1.3 ms Magnetic flux density: 1.5 mT Frequency: 75 Hz	Whole differentiation time (28 days)	Osteogenic	Increase in ALP activity Increase in OC expression Induction of ASC osteogenic differentiation Enhancement of matrix mineralization	Ongaro et al. 2014 [49]
BM-MSCs	Time of bursts: 4.5 ms Number of pulses: 20 Magnetic flux density: 1.6 mT (increase from 0 to 1.6 mT in 200 μs steps and then decrease to 0 mT in 25 μs steps during each pulse) Frequency: 15 Hz	8 h/day for 24 days	Osteogenic	Synergistic increase in ALP activity over that caused by BMP-2 Enhancement of the stimulatory effect of BMP-2 on OC	Schwartz et al. 2008 [40]
WJ-MSCs	Magnetic flux density: 1.8 or 3 mT Frequency: 75 Hz	8 h/day for up to 21 days	Chondrogenic	Increase in cell division Increase in cell densities Increase in COL II expression level Induction of early chondrogenic differentiation	Esposito et al. 2013 [36]
Sinusoidal PEMF					
ESCs	Magnetic flux density: 5 mT Frequency: 50 Hz	30 min/day for 14 days	–	Increase in proliferation rate	Bai et al. 2012 [32]
Low-frequency pulsed EMF (BEMER type)					
BM-MSCs/chondrocytes	Time of pulses: 30 ms Magnetic flux density: 35 μT (increase from 0 to 35 μT in 30 ms steps) Frequency: 30 Hz	Five times at 12-h intervals for 8 min	–	Impact on cell metabolism and cell matrix structure No increased expression of cancer-related genes	Walther et al. 2007 [48]
Pulsed EMF and single-pulse EMF					
ASCs	PEMF Time of bursts: 67.1 ms Number of pulses: 21 Time of pulses: 5.46 ms Magnetic flux density: 2 mT Frequency: 15 Hz SPEMF Time of bursts: 5 s Number of pulses: 30 Time of pulses: 5 ms Magnetic flux density: 1 T	PEMF: 8 h/day SPEMF: 3 min/day	Osteogenic/chondrogenic	No effects on cell viability Increase of the cartilaginous matrix deposition with both PEMF and SPEMF Enhancement of chondrogenic gene expression (SOX-9, COL II, and aggrecan) with both PEMF and SPEMF Enhancement of bone matrix gene expression (OC, COL I) only with PEMF	Chen et al. 2013 [42]

*ALP* alkaline phosphatase, *ASC* adipose tissue-derived mesenchymal stem cell, *BM-MSC* bone marrow-mesenchymal stem cell, *BMP* bone morphogenetic protein, *COL* collagen type, *CSC* cardiac stem cell, *ELF* extremely low frequency, *EMF* electromagnetic field, *ESC* epidermal stem cell, *IBSP* bone sialoprotein, *ICR* ion cyclotron resonance, *KDR* kinase domain receptor, *MAP2* mitogen activated protein 2, *MF* magnetic field, *MHC* myosin heavy chain, *MMP* matrix metalloproteinase, *ms* milliseconds, *MSC* mesenchymal stem cell, *NeuroD1* neurogenic differentiation 1, *NF-L* low-molecular weight neurofilament, *Nkx2.5* NK2 transcription factor related, locus 5, *OC* osteocalcin, *OPG* osteoprotegerin, *OPN* osteopontin, *OSX* osterix, *PEMF* pulsed electromagnetic field, *Runx* runt-related transcription factor, *SMA* smooth muscle actin, *SOX9* sex-determining region Y box 9, *SPEMF* single-pulse electromagnetic field, *Tau* microtubule associated protein tau, *TnI* troponin I, *VEGF* vascular endothelial growth factor, *WJ-MSC* Wharton’s jelly-mesenchymal stem cell

**Fig. 2:**
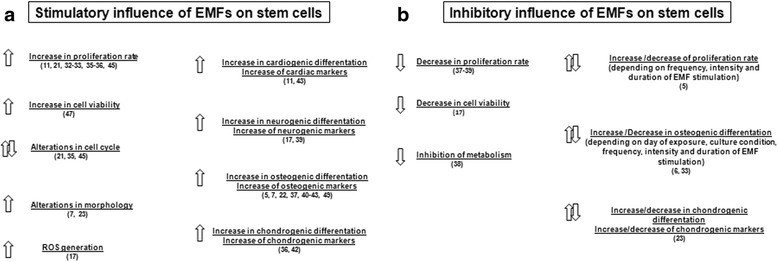
**a** Stimulatory influence and **b** inhibitory influence of EMFs on stem cells. *EMF* electromagnetic field, *ROS* reactive oxygen species

Endogenous electrical potentials and currents are generated in wounded tissues and they disappear when healing is complete. The EMF has a positive impact at different stages of healing (Fig. 2a). The processes affected by the EMF include cell migration and proliferation, expression of growth factors, nitric oxide signaling, cytokine modulation, and more. These effects have been observed using an EMF at low (30–300 kHz) and extremely low (3–30 Hz) frequencies.

## Effects of the EMF on stem cells during early development

Imprinting of maternal and paternal genetic components occurs during early development and epigenetic mechanisms are involved in this phenomenon. Importantly, disruption of imprinting may lead to abortion or disease (e.g., malformation, cancer). Endogenous EMFs are present in developing and regenerating tissues and organs, either in the extracellular space or in the cell cytoplasm. Their strength ranges from a few to several hundred millivolts per millimeter [24]. The EMF, together with diffusible chemical gradients, leads to polarization and formation of spatial patterns in the developing embryo, creating the signals necessary for correct placement of the components within the developing organism. Importantly, exogenous EMFs applied in vitro have been shown to influence cell behavior. The success rate of assisted reproductive technologies has been observed to be rather low in comparison with natural methods. In addition, the incidence of congenital malformations (Wiedemann syndrome, Angelman syndrome) is also higher in newborns conceived using assisted reproductive technologies compared with those conceived naturally [25, 26]. One of the reasons for the success rate decrease and malformation increase may be the exposure of stem cells in early embryonic development to the EMF during incubation before embryo implantation. Exposure to the EMF may disturb the normal imprinting process. The fact that the vast majority of cloned embryos die during embryonic development, despite their normal chromosomal complementation, suggests that epigenetic reprogramming in reconstructed oocytes is incomplete [27].

A body of evidence indicates that EMF affects the gene expression and differentiation process through epigenetic mechanisms [28, 29]. Chromatin modifications are involved in mediating the effects of EMF stimulation [30].

## Effects of the EMF on adult stem cells

### Effects of the EMF on stem cell proliferation and the cell cycle

**Fig. 3:**
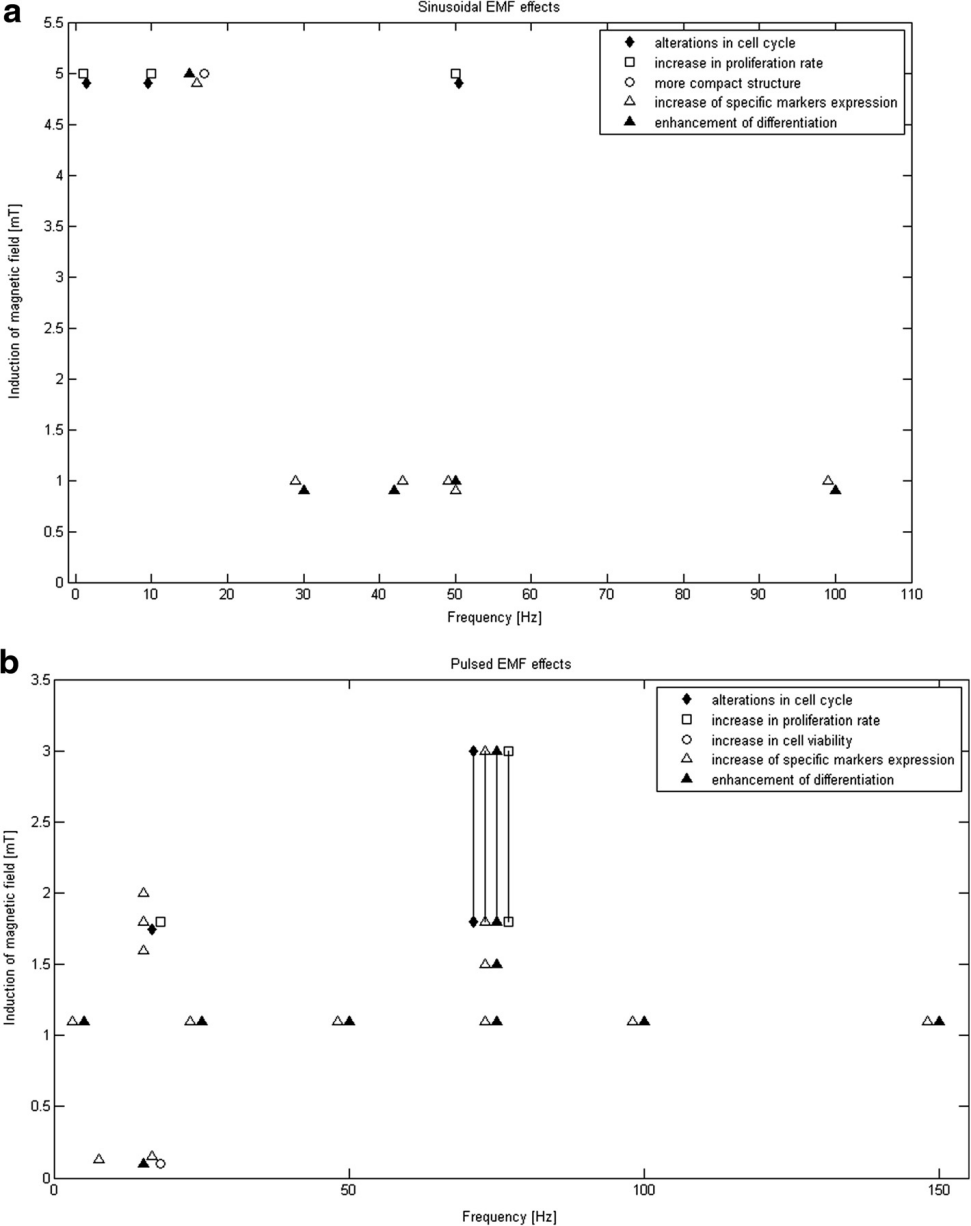
**a** Selected sinusoidal EMF effects on stem cell biology that occur with established parameters of both frequency and induction of magnetic field. Effects include: alterations in cell cycle [35]; increase in cell proliferation rate [35]; more compact structure [23]; increase in specific markers’ (neurogenic, osteogenic, chondrogenic) expression levels [6, 17, 23, 39]; and enhancement of differentiation (neurogenic, osteogenic, chondrogenic) [6, 17, 23]. **b** Selected pulsed EMF effects on stem cell biology that occur with established parameters of both frequency and induction of magnetic field. Effects include: alterations in cell cycle [21, 45]; increase in cell proliferation rate [21, 33, 36, 45]; increase in cell viability [47]; increase in specific markers’ (osteogenic, chondrogenic) expression levels [5, 7, 33, 36, 37, 40–42, 45, 49]; and enhancement of differentiation (osteogenic, chondrogenic) [7, 36, 41, 45, 49]. *EMF* electromagnetic field

**Fig. 4:**
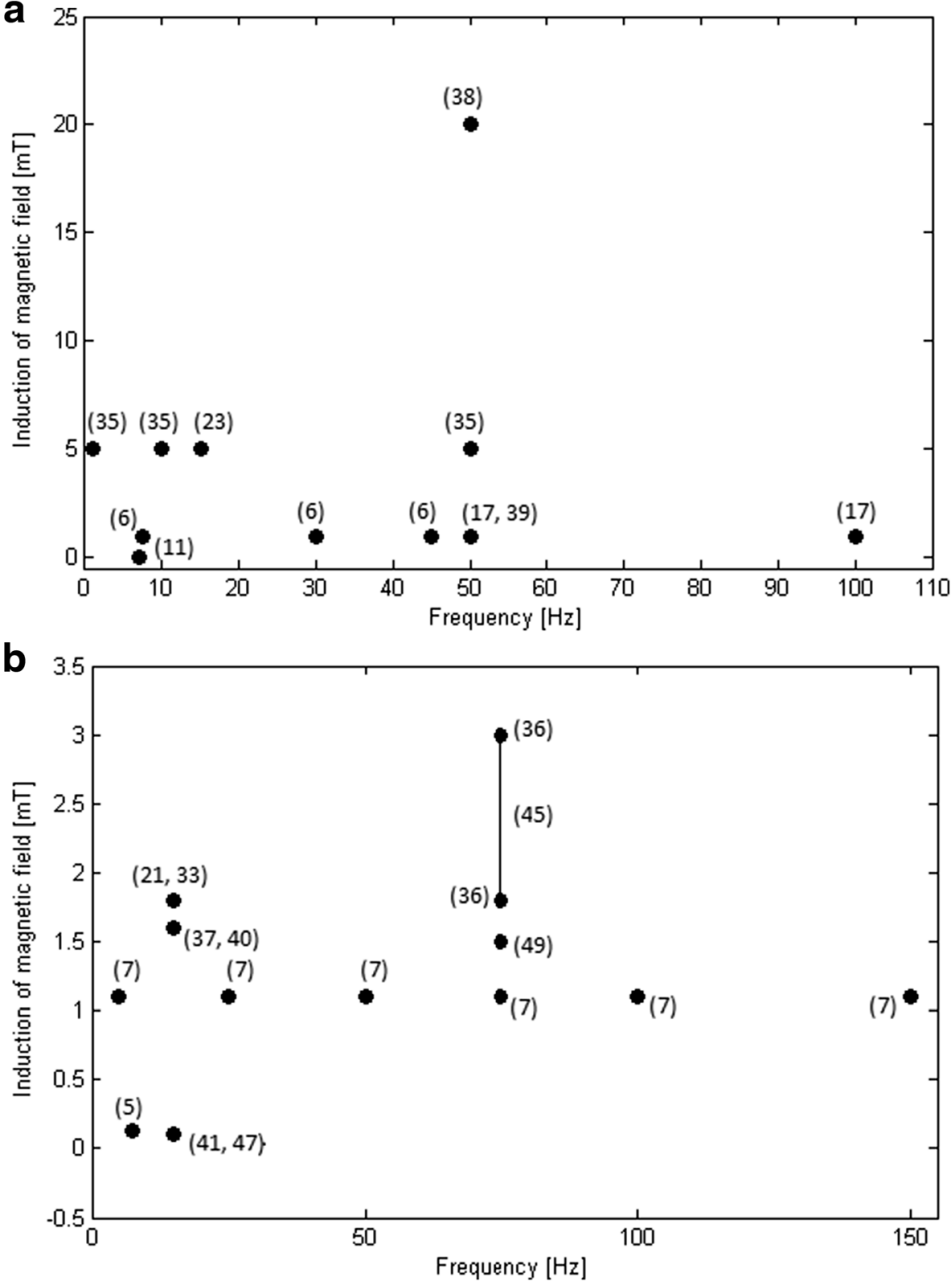
Parameters of **a** sinusoidal EMF and **b** pulsed EMF mostly used in current studies together with references

Scientific reports referring to the effects of the EMF on stem cell proliferation and the cell cycle have been inconsistent (Fig. 2a, b). Most research concerns human mesenchymal stem cells (MSCs). There have been numerous efforts to evaluate the effects of EMFs on different parameters; all of these are included and described precisely in Table 1. Consequently, we attempted to determine whether there is any general trend for selection of EMF characteristics and parameters in studies on human stem cell responses to EMF exposure (Fig. 3a, b). We gathered parameters of the EMF used in different studies for a sinusoidal EMF (Fig. 4a) and for a pulsed electromagnetic field (PEMF) (Fig. 4b).

For instance, several studies have demonstrated that the EMF (sinusoidal as well as pulsed) increases the stem cell proliferation rate [11, 31–33] (Fig. 2a). Interestingly, when murine stromal stem cells were exposed to an EMF, different cellular responses were noticed depending on the gender [31]. Further studies concerning the significance of donor gender in human adult stem cell behavior after EMF stimulation would therefore be interesting.

An increase in cell proliferation was observed when the cell culture was exposed to an EMF during the active proliferation stage [34]. Zhang et al. [35] showed that a sinusoidal EMF at 50 Hz caused the largest increase of human epidermal stem cell proliferation after 7 days of exposure (*p* < 0.05) compared with other experimental groups and an untreated group. Sun et al. [21] revealed that proliferation of bone marrow mesenchymal stem cells (BM-MSCs) treated with a PEMF began earlier compared with untreated cells. The enhancement of cell proliferation resulted in 20–60 % higher cell densities during the exponential growth phase. What is more, PEMF treatment of Wharton’s jelly mesenchymal stem cells triggered an increase in both cell division and cell density [36] (Table 1).

In contrast, Schwartz et al. [37] noted that PEMF treatment reduced the number of osteoblast-like cells cultured on a calcium phosphate surface by 40 %. It has also been reported that the EMF decreases the stem cell proliferation rate [38, 39] (Fig. 2b). However, we may suppose that the inhibition of MSC growth and metabolism is due to the higher EMF intensity value used by Yan et al. [38] in comparison with previous studies.

Tsai et al. [5] showed that PEMF stimulation did not alter proliferation of stem cells cultured in basal medium, while in osteogenic medium some differences occurred. There was a significant increase in cell density in the untreated group compared with the PEMF-treated groups at day 7 (75 %; *p* < 0.05), whereas at day 10 the PEMF-treated groups showed an increase in proliferation (62 %; *p* < 0.05), in contrast to the control group (Table 1).

Because of its influence on proliferation, EMF stimulation also affects the cell cycle. Zhang et al. [35] showed an increase in the percentage of cells in the S phase, representing the DNA synthesis stage, and a decrease in the percentage of cells in the G1 phase (*p* < 0.05). Moreover, these results were independent of the applied sinusoidal EMF frequency. Sun et al*.* [21] observed a 3–4 % (*p* < 0.05) increase in the proportion of cells in the G2/M phase during the first PEMF exposure and 4 h after the first PEMF stimulation. Then, 10 and 16 h after the first PEMF treatment, the percentage of cells in the G2/M phase and the S phase decreased by 8–12 % and 3–4 % (*p* < 0.05), respectively, whereas the proportion of cells in the G0/G1 phase, representing the newly divided cells, increased by 13–16 % (*p* < 0.05).

### Effects of the EMF on cell differentiation and marker expression

Numerous studies have been carried out on MSCs from different sources (Table 1). In most cases the differentiation was performed towards osteogenesis and chondrogenesis and the culture was grown in a medium containing differentiation factors. It has been reported that EMF stimulation affects the differentiation and the expression of specific markers (Table 1).

Many studies have shown the increase in osteogenic differentiation triggered by the EMF. Several studies have demonstrated an increase in alkaline phosphatase activity, an early marker of osteogenesis [5, 7, 33, 40]. Jansen et al. [41] observed higher expression levels of some osteogenic markers, such as bone morphogenetic protein BMP-2 (3.5-fold), transforming growth factor beta-1 (2.5-fold), matrix metalloproteinases MMP-1 (2.8-fold) and MMP-3 (2.1-fold), osteoprotegerin (1.7-fold), bone sialoprotein IBSP (twofold), and osteocalcin (OC; twofold). Interestingly, none of these markers was affected by a PEMF at the later stages of mineralization. Moreover, collagen type I (COL I) expression was steadily induced in the early stages of differentiation. In contrast, expression of receptor activator of NF-κB ligand (RANKL), which was insensitive to PEMF treatment in the early stages, was stimulated on day 14 (*p* < 0.05). Some investigations also showed higher expression of COL I and COL II, OC, runt-related transcription factor Runx2, and osterix in EMF-treated groups compared with control groups [5–7, 23, 33, 42, 43]. Moreover, studies performed by Creecy et al. [44] revealed that MSCs expressed both early (such as Runx2 and osterix) and late (osteopontin and OC) osteogenic genes as a function of level and duration of exposure to alternating electric current. The EMF stimulated matrix mineralization in comparison with untreated groups [6, 7, 33, 41].

The effect of the EMF depends on the external conditions of the cell culture. The EMF stimulated chondrogenic but not osteogenic differentiation when stem cells were cultured in a chondrogenic microenvironment. Some results suggest that the EMF affects the early stages of differentiation and reduces the time of differentiation [33, 36, 45].

Some studies have demonstrated alterations in neurogenic differentiation triggered by extremely low frequency (ELF)-EMF treatment. The expression of neural stem cell markers like nestin was thus decreased whereas neural cell markers such as mitogen-activated protein MAP2, neurogenic differentiation NeuroD1, low-molecular weight neurofilament NF-L, and microtubule-associated protein Tau were induced. Moreover, it was observed that the ELF-EMF accelerated the neural differentiation via reactive oxygen species (ROS)-induced epidermal growth factor receptor activation and, subsequently, the phosphorylation of Akt (known as protein kinase B) and cAMP response element-binding protein CREB. Based on these results, it has been suggested that EMF stimulation may induce neuronal differentiation without any chemicals or differentiation factors [17, 39]. Interestingly, Lee et al. [46] implied that ELF-EMF induces neural differentiation of BM-MSCs through activation of a ferritin-regulated mechanism.

The EMF has been reported to alter cardiac marker expression. Namely, troponin I, myosin heavy chain, connexin [43], and homeobox protein Nkx2.5 were upregulated (*p* < 0.05) by ELF-EMF treatment, tuned at the Ca^2+^ ion cyclotron energy resonance, compared with the untreated control. In contrast, vascular markers such as vascular endothelial growth factor and kinase domain receptor were downregulated or did not show any significant changes [11, 43].

However, we cannot clearly conclude how the EMF affects stem cell differentiation because the data concerning EMF stimulation of various markers’ expression are inconsistent. Some studies have revealed that the EMF may cause both an increase and decrease in proliferation and differentiation, depending on the day of exposure, cell culture conditions, or characteristics of the EMF, such as frequency, intensity, and time of stimulation [5, 6, 39] (Fig. 2b, Table 1).

### Other effects of the EMF on stem cells

EMF stimulation affects not only proliferation, the cell cycle, or differentiation of stem cells, but also other correlated processes. For instance, cells treated with ELF-EMF showed a tendency toward a more compact structure [23]. On the other hand, a PEMF changed the morphology of treated cells; stimulated cells were larger than control cells and became triangular and polygonal in shape, scales formed, and the cytoplasm contained abundant matrix and granular material compared with more immature untreated stem cells [7].

On the other hand, Hronik-Tupaj et al. [22] used alternating current electric fields for stimulation of BM-MSCs towards osteogenic differentiation. They observed upregulation of the stress markers heat shock proteins hsp27 and hsp70. Moreover, the increase in the hsp27 level was correlated with increased expression of lipofuscin, which is one of the aging or “wear-and-tear” pigments. These changes suggest a correlation between the expression of these markers and oxidative stress. They also observed higher levels of nicotinamide-adenine dinucleotide (NADH) and flavin-adenine dinucleotide and an increased redox ratio. Yan et al. [38] reported that ELF-EMF inhibits metabolism of treated MSCs.

## Mechanism of the EMF influence on stem cells

The mechanism of the EMF (sinusoidal as well as pulsed) influence remains unclear. The EMF affects a number of biological processes whose functions are closely related to the properties of the cell membrane. The EMF may act on membrane potential through hyperpolarization or depolarization. An ELF-EMF [11, 23] and a PEMF [21, 33] may also modify the transmembrane ion channels. Reorientation of some molecules causes deformation of ion channels and alters the ion flow, especially of Ca^2+^. Changes in intracellular Ca^2+^ levels affect the proliferation and differentiation of stem cells [6, 11]. The EMF may also influence signal transduction and intercellular communication [23].

Stem cells respond to the EMF differently depending on their state of differentiation. It is possible that the EMF (particularly PEMFs) modulates the activity of transcription factors and the level of cell cycle regulatory genes [33, 37, 40].

It is believed that one of the possible mechanisms involves the generation of ROS within the cell. Excessive concentration of ROS, such as superoxide anions (O_2_^–^) and hydrogen peroxide (H_2_O_2_), is considered to be cell destructive and results in inhibition of gene expression. In contrast, small amounts of ROS function as intracellular second messengers and activate signaling cascades involved in growth and differentiation of many cell types.

Some investigators imply that the ELF-EMF [17] and PEMF [37] act via a modification of signaling pathways, such as the extracellular signal regulated kinase pathway or phosphatidylinositol-4,5-bisphosphate 3-kinase/Akt signaling pathway. Park et al. [17] assumed that the ELF-EMF induced activation of NADH oxidase, which is involved in ROS production. The high level of ROS modifies signaling pathways by phosphorylation mechanisms.

Additionally, a weak EMF may accelerate electron transfer and thereby destabilize the hydrogen bonds of cellular macromolecules. This could explain the stimulation of transcription and protein expression, which has been observed after EMF exposure. However, the energy of a weak EMF is not sufficient to directly break a chemical bond in DNA. Therefore, it can be concluded that genotoxic effects are mediated by indirect mechanisms as microthermal processes, generation of ROS, or disturbance of DNA repair processes.

## Conclusions

Adult stem cells are very important within our body because they are responsible for homeostasis, regeneration, aging, and so forth. Stem cells may respond differently to external stimulation such as the EMF/PEMF depending on cell type, cell density, differentiation stage, and type of medium, as well as the characteristics of the EMF. So far we have few data on the influence of the EMF on stem cell biology. More studies are therefore required because stem cells are responsible for multiple processes within the human body, both desired (e.g., wound healing, regeneration) and undesired (e.g., pathological growth, carcinogenesis).

The parameters of EMFs (frequency, magnetic flux density) and times of exposure used by different research groups are quite diverse with no clear rationale for why particular parameters are chosen. We demonstrated the parameters and the ranges of parameters used in different studies for a sinusoidal EMF (Fig. 4a) and a PEMF (Fig. 4b). The successful use of sinusoidal EMFs in differentiation studies has mainly involved an EMF with parameters of 1–5 mT, 10–50 Hz. The only study using a sinusoidal EMF [38] in which a higher intensity of EMF was used (20 mT) did not show any significant effect on osteogenic differentiation. Additionally, the authors observed a decrease in MSC growth and metabolism. Importantly, we have to remember that higher intensities of the EMF may result in microthermal processes as well as the generation of eddy currents; therefore, besides the EMF, we have to take into account additional stimulatory factors*.* Additionally, we suppose that stress/oxidative stress may be a very important factor.

On the other hand, the most commonly used range of PEMF was 0.1–3 mT, 15–75 Hz. For example, there were two studies on osteogenic differentiation using very similar parameters (0.1 mT, 15 Hz) but with different pulse times: 5 μs [41] and 1 μs [47]. This difference in pulse times resulted in different osteogenic induction outcomes: an increase in differentiation [41] or no effect [47]. Thus, we may conclude that many factors may influence intracellular processes, such as the time of pulses, time of exposure, type of stem cells, or experimental methodology. It is worth noting that a wide range of EMF parameters have been used, depending on the desired effect. For instance, increases in cell proliferation were most evident at 5 mT, 50 Hz (for sinusoidal EMF), at 1.8 mT, 15 Hz (for PEMF), or at 1.8–3 mT, 75 Hz (for PEMF). In turn, the magnetic flux density used in most previous studies to enhance differentiation varied from 1 to 5 mT for sinusoidal EMF and from 0.1 to 3 mT for PEMF; the frequencies varied from 15 to 100 Hz for sinusoidal EMF and from 15 to 150 Hz for PEMF. This means that the aforementioned ranges of EMF parameters may be successfully used for stem cell-based therapies in which processes such as proliferation and differentiation are crucial. For example, the EMF has been shown to promote bone formation and therefore can be used in regenerative applications aimed at bone fracture healing [7]. Additionally, EMF stimulation of MSC chondrogenic potential during cartilage regeneration may result in beneficial effects [23]. What is more, EMF treatment can be used as an alternative tool for skin tissue engineering due to its positive impact on epidermal stem cell proliferation [32]. EMF modulation of stem cell differentiation into specific cell types promotes its application in cardiovascular disease [11] or neurodegenerative disorder [17] treatment.

Literature data concerning the influence of EMFs on stem cells with respect to carcinogenesis remain elusive. Defining the specific EMF range/characteristics inducing carcinogenesis would be very important. Walther et al. [48] did not observe any increase in cancer-related gene expression after low-frequency PEMF exposure. Radiofrequency EMFs have been suggested to trigger tumor promotion. However, the EMF mechanisms involved in induction of processes such as carcinogenesis and tumor formation are still under investigation and a lot of research needs to be done to explore this issue.

We hypothesize that some ranges of EMF parameters may promote regeneration but others may result in cancer formation, degeneration, and pathological alterations, depending on the stem cell type. These processes may be detected firstly at the epigenetic level, secondly at the genetic level, and finally at the proteomic and functional levels, leading towards either a positive or negative impact with respect to health and disease. To date, there are no data concerning this issue.

As a side comment, the number of cancer patients in our society is growing alarmingly. According to environmental health specialists, besides chemical pollution, this condition may be triggered by EMF exposure. Further studies are therefore required to explore this phenomenon at both in vitro and in vivo levels. We believe that EMF-based therapeutic applications may be used in the future for regenerative medicine approaches as well as in the “fight against cancer” or homeostasis restoration. More researchers, engineers, and medical doctors are required to improve the state of knowledge, working on stem cell biology, stem cell transplantation, biophysics, biochemistry, tissue engineering, engineering, regenerative medicine, oncology, and other areas to explore this phenomenon.

In conclusion, properly adjusted values of EMF frequencies, times of stimulation, as well as the microenvironmental niche may affect EMFs’ impact on stem cell proliferation, differentiation, and migration to result in the desired therapeutic outcome. Additionally, this knowledge may help us to determine the best approach for using properly adjusted EMFs in future autologous stem cell-based therapy. Importantly, it is reasonable to check the impact of the EMF with respect to carcinogenesis.

## Abbreviations

ASC: adipose-derived mesenchymal stem cell
BM-MSC: bone marrow-mesenchymal stem cell
COL: collagen
ELF: extremely low frequency
EMF: electromagnetic field
MSC: mesenchymal stem cell
OC: osteocalcin
PEMF: pulsed electromagnetic field
ROS: reactive oxygen species
